# The healthy human microbiome

**DOI:** 10.1186/s13073-016-0307-y

**Published:** 2016-04-27

**Authors:** Jason Lloyd-Price, Galeb Abu-Ali, Curtis Huttenhower

**Affiliations:** Biostatistics Department, Harvard School of Public Health, Boston, MA 02115 USA; Microbial Systems and Communities, Genome Sequencing and Analysis Program, The Broad Institute, Cambridge, MA 02142 USA

## Abstract

Humans are virtually identical in their genetic makeup, yet the small differences in our DNA give rise to tremendous phenotypic diversity across the human population. By contrast, the metagenome of the human microbiome—the total DNA content of microbes inhabiting our bodies—is quite a bit more variable, with only a third of its constituent genes found in a majority of healthy individuals. Understanding this variability in the “healthy microbiome” has thus been a major challenge in microbiome research, dating back at least to the 1960s, continuing through the Human Microbiome Project and beyond. Cataloguing the necessary and sufficient sets of microbiome features that support health, and the normal ranges of these features in healthy populations, is an essential first step to identifying and correcting microbial configurations that are implicated in disease. Toward this goal, several population-scale studies have documented the ranges and diversity of both taxonomic compositions and functional potentials normally observed in the microbiomes of healthy populations, along with possible driving factors such as geography, diet, and lifestyle. Here, we review several definitions of a ‘healthy microbiome’ that have emerged, the current understanding of the ranges of healthy microbial diversity, and gaps such as the characterization of molecular function and the development of ecological therapies to be addressed in the future.

## Background

Humans have co-evolved with the trillions of microbes that inhabit our bodies and that create complex, body–habitat-specific, adaptive ecosystems that are finely attuned to relentlessly changing host physiology. Dysbioses in the microbiome have been associated with numerous diseases, including inflammatory bowel disease, multiple sclerosis, diabetes (types 1 and 2), allergies, asthma, autism, and cancer [1–5]. Like the concept of the pathogenicity of a single microbial taxon, dysbiosis of a microbial community can be difficult to define but could be considered as a perturbation that departs from an otherwise balanced ecology [1] to prolong, exacerbate, or induce a detrimental health effect. Thus, finding features that broadly distinguish healthy from unhealthy microbiomes will aid in the diagnosis of microbiome-related diseases and could potentially provide new means to prevent disease onset or to improve prognosis. Many potential features common to healthy microbiomes have been proposed, including prevalent organisms or molecular pathways [6] as well as norms of certain ecological properties, such as diversity or stability [7, 8]. Microbiomes regularly show a large degree of interpersonal diversity even in the absence of disease [7, 9]. This complicates the identification of simple microbial constituents or imbalances that either cause disease or reflect a diseased state. An understanding of the properties of a healthy microbiome, and the many different microbial ecologies that are encountered in the absence of overt disease, is therefore a necessary first step to identifying and correcting microbial configurations that are implicated in disease.

In this review, we use “healthy” to refer to the absence of any overt disease (as defined in [10], unless otherwise specified for particular studies). Most available data describe the gut microbiome and so many of the findings discussed here are from this area, though most principles apply to microbial habitats throughout the body. Early research into the ecology of the microbiome sought to identify a “core” set of microbial taxa universally present in healthy individuals who lack overt disease phenotypes, under the hypothesis that the absence of such microbes would indicate dysbiosis [11]; but studies of ecological diversity among healthy individuals revealed sufficient variation in the taxonomic composition of the microbiome to rapidly render such a hypothesis unlikely [11, 12]. Even shared taxa, from individual species to entire phyla, were found to vary in abundance by more than an order of magnitude among healthy individuals [7, 11]. Characterizing a “healthy” microbiome as an ideal set of specific microbes is therefore no longer a practical definition [2, 6].

An alternative hypothesis is that of a healthy “functional core”: a complement of metabolic and other molecular functions that are performed by the microbiome within a particular habitat but are not necessarily provided by the same organisms in different people [6]. Such a core might need to be present as genetic potential (that is, encoded within DNA metagenomes) much as the human genome must not encode serious deleterious mutations to be healthy or it may need to be expressed and well-regulated within an individual for him/her to remain healthy (that is, it must be encoded by RNA metatranscriptomes or present in the form of protein or small molecule products), or of course a combination thereof. The functional core must, of course, include at least the housekeeping functions necessary for individual microbial life, which must be present genomically and correctly expressed; interestingly, these properties may also include functions specific to microbes’ niches in the human ecosystem. Such functions may include processes that are not carried out by human cells and thus represent a potential basis for symbiotic host–microbial relationships. A healthy microbiome may be characterized further by its behavior over time [2, 8]; intuitively, a health-associated microbiome must have a degree of resilience to external (for example, dietary or pharmaceutical) or internal (for example, age- or stochastic-drift-related) changes. Even if a particular community structure provided all necessary core functions, without this resilience it could not guarantee these functions for long. Thus, the resistance of a microbiome to stress and perturbation and its ability to recover to a healthy functional profile afterwards are among the potential properties that characterize a healthy microbiome [2, 13].

Here, we review the current characterization of the healthy microbiome in terms of the normal microbial residents and their core functions, ecological properties, and temporal dynamics. We conclude by identifying key outstanding questions and research directions in this field and speculate on their solutions and impact. A combination of recent technological advances and activity within the field has driven a surge of interest in the human microbiome in health and disease (Table 1) and thus this review aims to summarize the variety of current perspectives on what may constitute a healthy microbiome.

**Table 1:** Diversity of recent microbiome research, which has focused mainly on the gut

	Publications	
Terms	All	2011–2016
Gut | colon | intestinal	17,546	10,707
0ral | mouth | tongue | tooth | subgingival | supragingival	4843	2089
Urogenital | vaginal | penile	1477	706
Skin | cutaneous	1372	754
Airway | lung	764	524
Placenta | breast milk	702	426
Ocular | eye	152	82

Number of results obtained by searching for “(microbiome | microbiota | microflora) (<Terms>)” on PubMed (retrieved 31 March 2016)

## Our evolving understanding of the healthy microbiome

Early studies sought to identify the normal set of microbes that colonize healthy people, primarily in the gut, by culture and characterization of physiological properties. Such studies best highlight organisms that grow well in the lab environment, such as *Escherichia coli*. This bias led to the perception that *E. coli* is an abundant and prevalent member of the human gut microbiome [14]. The introduction of strictly anaerobic techniques in the 1970s allowed the recovery of more than 300 bacterial species from the gut alone [15]; furthermore, the counting of viable cells within standardized serial dilutions in selective media permitted quantification of these species. A summary of four large studies from this era [12] looking at stool samples from 141 Americans on different diets found that bacteria of the genus *Bacteroides* and anaerobic cocci were both prevalent and abundant, whereas the genus *Clostridium* was ubiquitous in lower abundance, though no single species (as then defined) was observed in all subjects. Other prevalent but lower-abundance bacteria included members of the genera *Bifidobacterium*, *Eubacterium*, *Lactobacillus*, and *Streptococcus*, as well as facultative anaerobes such as *Escherichia*.

It was already suspected at this time that a large number of human-associated microbial species remained undiscovered, with one study estimating the simultaneous presence of some 400 microbial species in a healthy colon [16, 17]. However, the fastidious requirements of some microbes and the labor-intensive nature of the work required to culture them presented a significant barrier to their discovery [12]. Further, not all microbes can be well-distinguished as species or strains by culturing on selective media alone; for example, the different high-abundance *Bacteroides* species are particularly difficult to disentangle [12, 17]. In addition, such studies of community composition were even more difficult to extend to non-bacterial microbes, such as viruses and fungi, and were even more impractical for studies of body habitats that are less microbially rich than the gut. New methods were required to study these aspects of the healthy microbiome.

Culture-independent techniques such as DNA sequencing [18] and fluorescence in situ hybridization (FISH) [19] are now widespread and their democratization has allowed the DNA content of microbial samples to be interrogated directly [20]. Early studies using FISH targeting the 16S ribosomal RNA gene suggested that at least two-thirds of the gut bacteria in a western European cohort could be attributed to a set of six groups at approximately the species/genus level: two *Bacteroides*, two *Clostridium*, *Streptococcus*/*Lactococcus*, and *Eubacterium rectale* [19]. This has since proved to be optimistic and, even at the time, large variability was observed in the abundances of these groups between samples (standard deviations of ~60–80 % of their means) [19].

Some of the earliest efforts to sequence 16S rRNA genes directly from samples showed that 85–95 % of bacterial abundance corresponding to known species could be attributed to three bacterial groups related to *Bacteroides*, *Clostridium* cluster XIVa, and *Clostridium* cluster IV [21, 22]. 16S studies also showed a large diversity in the taxonomic composition both between healthy people and among closely linked biogeographical sites within a single person (such as mucosal and stool samples [23]). However, in all of these studies, the majority (75–80 %) of sequence clusters did not match any documented species at the time [21–23], explaining much of the underestimation of diversity in previous work.

The advent of massively parallel shotgun sequencing (high-throughput sequencing technologies) has substantially resolved the taxonomic composition of this microbial “dark matter” [24], although a striking percentage of functional diversity remains to be characterized (up to 50 % [25]) as does the composition of non-reference populations (discussed below). Initial findings echoed the large interpersonal differences, even between twins [26], but also implied the existence of a set of microbial genes that are common to all individuals [26, 27]. This helped seed the model that, like conserved housekeeping genes in individual organisms, a “core microbiome” can be defined at the functional rather than at the taxonomic level [26, 27].

## Population-scale baseline cohorts

Large-scale projects have since been launched to characterize the diversity of microbial composition and its functional potential, building on the still-increasing throughput and cost-effectiveness of sequencing and other molecular assays. In 2010, the Metagenomes of the Human Intestinal Tract (MetaHIT) study reported gut metagenomes from stool samples from a cohort of 124 European adults (predominantly ‘healthy’), which at the time exceeded the sequencing volume of all previous microbiome studies by almost 200-fold [9]. In 2012, the Human Microbiome Project (HMP) reported the results of 16S profiling on 242 healthy adults from the United States and metagenomic sequencing on a subset of 139 individuals, with samples representing 18 body habitats distributed between five major body areas [7]. A large Chinese study on type 2 diabetes soon contributed an additional 145 gut metagenomes, approximately half of which were from non-diabetic controls [28]. Further, the MetaHIT consortium has since continued to publish new gut metagenomes from European adults [29–31]. Altogether, the number of population-scale healthy microbiomes surveyed in the gut and other body sites now exceeds 2000 individuals spanning multiple continents.

## Typical components and diversity of the microbiome

### Bacterial components of a healthy microbiome

**Fig. 1:**
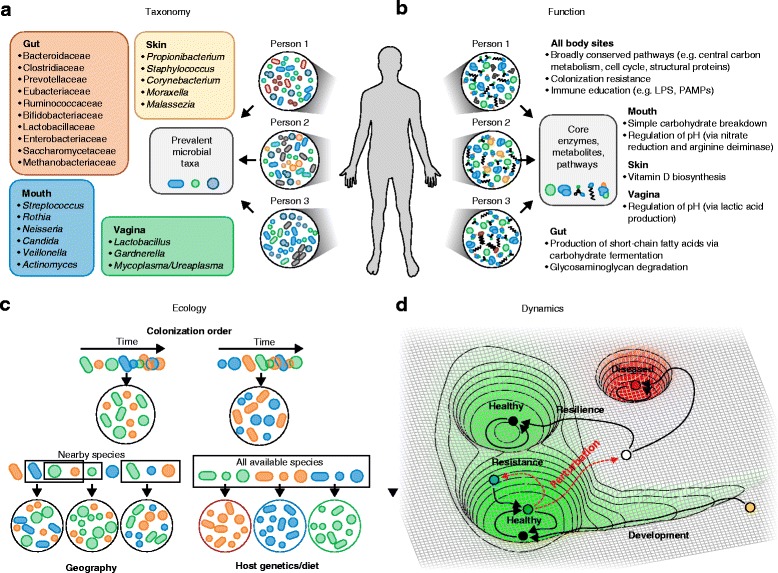
Possible definitions of a healthy microbiome: composition, function, dynamics, and ecology. **a** Early definitions of a “healthy” microbiome generally focused on sets of taxa that might be expected to be found prevalently in healthy people. While purely taxonomic cores of any type have remained elusive, even in relatively narrowly defined populations, each body-site habitat possesses strong phylogenetic enrichments. Typical genera (or families in the gut) in healthy populations at different sites are shown here [7, 9, 15, 33–35]. **b** Metagenomic measurements have allowed the functional potential of the microbiome at different sites to be assessed. These studies have yielded more consistently shared functional cores of body-wide and niche-specific pathways that are maintained in health [6, 7, 9, 98]. *LPS* lipopolysaccharide, *PAMP* pathogen-associated molecular pattern. **c** Ecological assembly patterns provide another possible definition of a healthy microbiome, because each host may draw from a “typical” meta-population of potential microbes through a mix of partially stochastic processes. These processes may include the order in which microbes colonize their respective human habitat (affected by geography and early exposures, for example), the prolonged availability of each microbe in the host’s local environment, and host selection (through diet or genetics, adapted from Fig. 1 of [101]). **d** The healthy microbiome can also be characterized in terms of its dynamics, depicted here in a simplified model as a conceptual energy landscape. The infant microbiome (*yellow point*) starts out in an unstable state and gradually descends towards one of potentially several healthy adult attractor states. Perturbations (*dashed red arrows*) can either be resisted (*green point*) or can move the microbiome out of the healthy state, after which a resilient microbiome will return to a healthy state (not necessarily the original healthy state) or fall into an unhealthy state (*red*)

The ecosystem of the colon has been the most intensively studied body habitat (Table 1) as it boasts a remarkable diversity between people and a microbial biomass (cell count) that eclipses that of other body sites by more than an order of magnitude [32]. In combination with the early rise of 16S rRNA gene sequencing and anaerobic culture techniques, these properties of the gut have led to a particularly strong focus in the literature on bacterial gut microbiome residents. Over 1000 gut bacterial species have now been characterized [15], providing a significant “parts list” of bacterial constituents. Interestingly, molecular phylogenetics has led to the reclassification of many of these species in the past 20 years. Of particular interest, species within *Bacteroides*, previously considered the most prevalent and abundant bacterial genus in the gut, have been reclassified into five genera: *Alistipes*, *Prevotella*, *Paraprevotella*, *Parabacteroides*, and *Odoribacter*, with additional culture-based and culture-independent molecular work ongoing [15]. An estimated 1000–1150 bacterial species were prevalent in the MetaHIT cohort’s gut microbiomes, of which each person carried ~160 species on average [9]. Healthy gut microbiomes as assessed by sequencing are consistently dominated by bacteria of two phyla—Bacteroidetes and Firmicutes [7, 9]—though even when considering this broad level of classification, individuals vary by more than an order of magnitude in their Firmicutes/Bacteroidetes ratios [7]. Prevalent bacteria in feces that have been identified through molecular techniques have broadened the lists above to include bacteria from at least eight families (Fig. 1a).

Although less well-studied than the gut, many other body habitats within healthy individuals are occupied by microbial communities [7]. Community composition is more similar within than between habitats (for example, oral communities share greater similarity with oral communities in other people than with other habitats within the same person), although, in turn, inter-individual differences within habitats are much greater than intra-individual variability over time [7]. Oral sites harbor particularly diverse microbiomes [33], similar in complexity to the microbiome of the gut [7], and tend to be dominated by *Streptococcus* spp. [7]. Skin sites differ primarily with the local properties of the skin (dry versus moist versus sebaceous [34]) and are colonized primarily by *Corynebacterium*, *Propionibacterium*, and *Staphylococcus* [34]. The healthy vagina contains one of the most remarkably structured microbial ecosystems, with at least five reproducible community types, or “community state types”, each dominated by a single species of *Lactobacillus* (*L. crispatus*, *L. iners*, *L. jensenii*, or *L. gasseri*) or by a mixture of other microbes including *Gardnerella* [35, 36]. Significant determinants of a woman’s community state type include race/ethnicity [35, 37] and pregnancy [37], although even in this structured ecosystem within-subject longitudinal variation is substantial and, to date, has no fully explained causes.

Several significant body habitats tend to have particularly low microbial biomass in healthy individuals and are thus more difficult to characterize. The lung, for example, is near-sterile in the absence of infection or chronic disease, leading to great interest in identifying its normal residents but also to substantial technical challenges in sampling and sequencing the site [38–40]. Likewise, breast milk [41] and the placenta [42] are of interest for the early establishment of both a healthy microbiome and the potential circulating blood [43] or tissue [44, 45] microbiomes for normal immune control of opportunists. There are considerable difficulties in acquiring metagenomes from such environments and thus most studies have relied on contamination-sensitive amplicon surveys [46] and relatively low-throughput single-cell techniques, such as FISH or microfluidics. Larger-scale carefully controlled studies are thus needed to establish the functionality of these challenging low-density microbial habitats.

### Archaea, viruses, fungi, and other eukaryotes

The study of the healthy microbiome has been greatly enriched for bacteria [7, 9], with less attention given to other microbial domains. The human microbiome, though, spans the tree of life and thus includes archaea, viruses, and eukaryotes. A small number of archaeal genera have been identified in the healthy human microbiome, primarily in the gut. Species of the *Methanobrevibacter* genus are the most prevalent [47] in the gut, with their status as “healthy” members of other body sites’ communities remaining somewhat unclear [48]. *Methanobrevibacter smithii* in particular has been found to be well-adapted to the human gut, optimizing the digestion of dietary polysaccharides by other microbes [49] and adapting its gene expression in the presence of common gut bacteria such as *Bacteroides thetaiotaomicron* [49]. The human virome is particularly extensive and, while under-characterized, is recognized as an integral part of the healthy human ecosystem [50]. With the hypervariable nature of viruses, each person is expected to harbor a unique virome [51, 52], consisting primarily of bacteriophages [50] (an estimated 5 % of the gut bacterial gene complement codes for prophage proteins [9]). Phages also provide an additional means of horizontal gene transfer among otherwise distantly related bacteria [53]. As molecular-profiling techniques for archaea, viruses, and eukaryotes are still less well-developed than those for bacteria (even those using culture-independent approaches [47, 54]), information on the molecular functionality of these organisms within in situ communities remains limited.

Although the best-known eukaryotic microorganisms found in or on the human body (principally fungi and protists) are typically pathogens, it is important to remember that many such eukaryotes, in particular *Candida*, *Malassezia*, and *Saccharomyces*, are pervasive even in healthy populations [55–58]. Trans-kingdom interactions are responsible for at least part of the ecological and immune balance of the healthy microbiome; for example, there is apparent competition between bacteria and fungi across skin biochemical environments [59] or in *Lactobacillus* control of fungi in the gut [55] and vagina [60]. Although few examples exist, direct mutualistic relationships between humans and fungi have been found, of which the best-characterized involves the probiotic yeast *Saccharomyces boulardii*, originally isolated to combat cholera [61]. Some protozoa are even common inhabitants of healthy microbiomes [58, 62], albeit (like viruses) with even greater interpersonal variability than bacteria [58]. Further, the presence of some protozoa, such as the common *Blastocystis*, has been associated with reduced risk of gastrointestinal disease [63]. Finally, although multicellular eukaryotes such as helminths have generally been eliminated from gut microbiomes in Western cultures, they have been a component of the gut microbiome for a significant portion of our recent evolutionary history [64]. Given their potent immunomodulatory capabilities and interactions with the other inhabitants of the normal gut microbiome (such as *Lactobacilli* [65]), their elimination may have removed an important educator of our immune systems [64].

### Geographical variation in the healthy microbiome

Studies contrasting the gut microbiomes from different countries have identified systematic differences in microbial composition, although it remains challenging to tease apart inter-batch technical effects from inter-population biology. Comparison between the largest cohorts from three continents—MetaHIT (European), HMP (American), and Chinese diabetes cohorts—found that the inter-country variation in taxonomic composition significantly exceeded inter-personal variation, which was not solely attributable to technical differences in experimental methodologies [29]. Nevertheless, smaller international studies have also identified geography as one of the major sources of large-scale variation in the microbiome, including between North and South America [66], Europe and Africa [67], Korea and Japan [68], and between rural and urban populations of Russia [69] and China [70]. Among possible drivers of this variation, diet has been suggested as an important contributor [67], along with other factors including geography, early-life exposures, and genetics [29, 71]. No one study has yet shown any of these factors to be causal in the large observed inter-population differences in healthy microbiomes [72].

Geographic differences at the strain level are also of interest, particularly as strain signatures exhibit greater temporal stability than do microbial abundance profiles [8, 73, 74]. Research in this area is preliminary but shows that strain differences are not particularly pronounced between countries or continents. Species such as *Bacteroides coprocola* and *Prevotella copri* show the greatest differences [73] and strain-level variants in antibiotic resistance genes spanning populations [75]. Strain-level microbial forensics on highly heritable species such as *Helicobacter pylori* have been remarkably insightful in tracing historical effects on the microbiome [76, 77] and culture-independent techniques should be leveraged for thorough large-scale population surveys in the future.

## Microbiome establishment and early colonization

Factors that influence early-life microbiome dynamics are important precipitators of a healthy microbiome. Microbial introduction and persistence is a semi-stochastic process influenced by many elements (Fig. 1c), yielding a healthy adult-like configuration only after the first few years of life [66, 78–80]. Enrichment of the infant gut microbiome for symbionts such as *Bacteroides*, *Parabacteroides*, *Clostridium*, *Lactobacillus*, *Bifidobacterium*, and *Faecalibacterium prausnitzii* provides several determinants of a healthy microbiome. Once established, these are the main producers of short chain fatty acids (SCFAs), an important source of energy from non-digestible carbohydrates [81]. SCFAs are immunomodulatory [82], inhibit common pathogens, and are hypothesized to possess tumor-suppressive properties [83, 84]. The gut microbiome is an inextricable requirement for immune system education and the establishment of these beneficial genera early in life promotes immune tolerance and can consequently attenuate or abrogate autoimmune diseases [1, 85–89].

Delivery mode can affect early-life establishment of microbiota such that Caesarean section is associated with enrichment for opportunists, including *Haemophilus* spp., *Enterobacter cancerogenus*/*E. hormaechei*, *Veillonella dispar*/*V. parvula* [78], and *Staphylococcus* [80]. These microbes continue to persist at least throughout the first year of life [78] and possibly contribute to infant infection burden. Diet also represents a strong selective pressure on the microbiome [71, 90] and breast-feeding (as the first diet) favors certain microbial clades from among the initial microbiota that may have assembled at random. For example, human milk oligosaccharides (HMO) can be used as the sole carbon source by only a handful of *Bifidobacterium* and *Bacteroides* species [91] and, more so, bovine milk oligosaccharides (BMO) were recently shown to promote growth and metabolism in a microbiota-dependent manner in animal infant models [92]. While this model may not translate directly to human infants because of the unique structural diversity, complexity, and high concentration of HMO [93, 94], it lends further support to the inference that the long-term benefits of breast-feeding [95] are mediated, in part, by the microbiome.

## Hallmarks of health

### Functional core

While large interpersonal differences are observed in the taxonomic composition of the microbiome at all sites, the abundance of metabolic pathways is considerably more consistent across people for a given site [7, 9, 26, 27]. Further, while the composition of the microbiome changes drastically over the first years of life, this functional profile is established early on and remains stable thereafter, at least in the gut [72]. This suggests that one definition of a “core” healthy microbiome might include specific microbial gene family combinations, metabolic modules, and regulatory pathways that together promote a stable host-associated ecology [96, 97]. This core includes functions from at least three groups: first, and most simply, the housekeeping functions necessary for all microbial life, such as transcription and translation, energy production, and structural components [6, 7, 9]. Second, this core includes processes that are specific to human-associated microbiomes across body-site habitats, such as adhesion to host cell surfaces and the production of compounds implicated in host–microbe interaction (including essential vitamins, such as vitamin K, and immunostimulatory compounds) [6, 7]. Finally, different body habitats each have their own specialized core functions [98]. For example, in the gut, core functions include glycosaminoglycan biodegradation, the production of several short-chain fatty acids, enrichment for specific lipopolysaccharides, and the production of vitamins and essential amino acids [6, 9, 98, 99] (Fig. 1b). Which of these functions tend to be enriched in a given population can be affected by long-term selective pressures such as diet [67]. A necessary condition for a healthy microbiome is therefore the presence of an assemblage of microbial species that can carry out specific sets of biomolecular functions in each of the niche-specific biochemical environments across the body.

### Healthy community ecology

If microbial communities assemble on the basis of their coverage of a core set of functions while selecting from a large meta-population of potential colonizers, they are likely to be ecologically diverse [100–102], both in terms of richness (number of taxa present) and evenness (abundance of many microbial constituents). High diversity has been generally associated with health [11] and temporal stability [103]. The latter could, for example, be the result of the increased functional redundancy that comes with a more diverse set of microbes, even if the functional potential of the assembly is minimally achievable with fewer taxa. Conversely, a relative lack of diversity is apparent in the gut microbiome in diseases ranging from obesity [26] to inflammatory bowel disease [104] and types 1 [72] and 2 [28] diabetes; and in the skin microbiome in atopic dermatitis [105] and psoriasis [106]. Antibiotics also cause a drastic reduction in the diversity of the microbiome with highly variable recovery dynamics [107], potentially weakening the community’s ability to exclude pathogens. This may clear the way for infection by pathobionts—normal microbial community members that become detrimental under perturbation, such as *Candida albicans* [57]. The principle that high diversity is “healthy” does not hold for all body sites, however, as diversity in the vaginal microbiome can be associated with bacterial vaginosis [108], cervical intraepithelial neoplasia [109] (an abnormal growth on the cervix), pre-term birth [36], and inflammation [110].

Given the typical observation of increased microbiome diversity in health, it has been hypothesized [111] that developed countries’ consistently reduced gut microbial diversities may account for higher chronic disease rates relative to those seen in developing countries and primitive societies [66, 112, 113], termed the “disappearing microbiome hypothesis” [111]. This loss of diversity may be linked to a high-fat, high-refined-sugar, and low-fiber diet [114]. Humanized mice on such a diet exhibit a depletion in microbial diversity [114] and though this is recoverable by returning to a high-fiber diet within a generation, it becomes fixed after four generations [114]. If this result generalizes to human populations, it increases the urgency of developing rationally targeted microbiome maintenance or therapeutic methods, so as to steer less health-promoting microbiomes towards more natural assemblages. The disappearing microbiome hypothesis in some ways represents an evolution of the “hygiene” or “old friends” hypotheses [115], all of which suggest that while modern North American or European cohorts may represent “healthy” microbiomes, their relationship to what is evolutionarily “normal” may be more complex.

### Resistance, resilience, and stability

Other hallmarks of health from the microbial ecology perspective are the ability to resist perturbation (which might result from the entry of a pathogen, alteration of diet, or medication) and to return to a healthy state afterwards. These properties have been termed resistance and resilience, respectively [2]. For example, after an antibiotic treatment, healthy gut communities generally recover to their previous state after a few weeks to months [116]. A recent definition of microbial health thus explicitly comprises not a single static state but rather a dynamic equilibrium [2]. In this view, a healthy microbiome corresponds to an attractor of an underlying dynamic system (Fig. 1d), in a similar manner to cell fate in a metazoan [117]. Attractors capture both resistance and resilience, in that the system will resist a departure from an attractor, and unless a fluctuation (which might be due to external perturbation or internal stochasticity) is sufficiently large, it will tend to return to the steady state area [117]. The most visible examples in the human microbiome may be transitions between community state types in the healthy vagina; although their specific health implications are not yet enumerated, not all community state types have the same degree of stability [36]. The gut microbiome is also in flux, gaining and losing species over time, with different taxa having different stabilities and with some consistently remaining in the gut for many years [8]. The mechanisms by which specific taxa persist are not yet well-delineated, but it is interesting to speculate whether such mechanisms might relate to the driving principles behind the assembly of the microbiome. If specific communities do assemble primarily to fill a suite of habitat-suited functional niches [6], then species that provide key metabolic, signaling, immunomodulatory, or other roles in a particular assembly may be more temporally stable than those in the functional periphery. Coupling dynamics with the taxonomic diversity and immense molecular functional potential of the microbiome is thus a reminder of the human microbiome’s complexity and, as a result, the difficulty of defining even the apparently simple concept of microbial health.

## Outlook

The era of population-scale whole-microbiome epidemiology has only recently begun, with the HMP [7, 118] and MetaHIT [9, 29] among the first large cohorts to include broad reference data in health, and several more cohorts soon to come. Data to date have been dominated by cross-sectional, amplicon-based studies of Western populations, all of which are efficient and accessible but which do not yet paint a consistent, comprehensive picture of the global, dynamic, healthy microbiome. Large-scale epidemiology in other areas of human health, such as nutrition and lifestyle, has built a solid foundation for prospective, long-running cohorts, painstaking analyses, and carefully validated measurement instruments [119–121], all of which represent particularly promising avenues of exploration for the microbiome. Nesting longitudinal microbiome studies in existing cohorts has the advantage of utilizing long-term collected lifestyle, dietary, medical, and phenotype information, as well as integration with banked biospecimens. An example of an unconventional large-scale study, notable for its infrastructure and outreach, is the American Gut project: a crowd-funded source of microbiome reference data paired with subject-provided environmental metadata. Prospective studies with detailed molecular data, while more expensive and logistically challenging, will also be necessary to facilitate predictive models and to establish the causality of dysbioses. The ongoing “HMP2” or Integrative Human Microbiome Project (iHMP) [122] includes three such longitudinal studies, which are providing multi-omic data for health and chronic disease, along with protocols and computational tools as a foundation for future work.

While many current studies of the microbiome focus on disease, a better understanding of the healthy microbiome will itself help to develop new microbial community diagnostics and therapeutics [123]. To the degree that universal features of the healthy microbiome can be defined, their absence may be predictive of disease onset generally, much like the presence of features specific to any one condition’s dysbiosis (especially useful if it occurs prior to disease onset). Alternatively, personalized medicine and longitudinal monitoring may serve the same purpose with respect to departure from an individual’s own “healthy” state [1, 104]. Therapeutically, as targeted interventions are developed to manipulate the microbiome, the treatment of a dysbiosis need not return to *the* healthy state from which an individual departed (due to a perturbation such as antibiotic treatment or the invasion of a pathogen), but perhaps only to *a* healthy state (Fig. 1d). Likewise, even if a microbial dysbiosis proves to be responsive rather than causal in any given disease state, the return to a “healthy” state may still provide therapeutic benefit [73, 101, 124].

One of the biggest outstanding gaps in understanding the basic biology of the “healthy” microbiome is perhaps at the level of annotating its molecular function: up to 50 % of microbial gene families encountered in the human microbiome remain functionally uncharacterized, even in well-studied environments such as the gut [9, 25, 29]. This is to a degree true in individual microbial isolate genomes as well, where even the well-studied *E. coli* K12 contains some 18 % of gene products with no reported function [125], with appreciably more at the *E. coli* species pangenome level [126]. It is likely, for example, that some of these genes are responsible for microbe–microbe or host–microbe interactions and thus will only be expressed or characterizable in community settings. Population-scale studies of the microbiome can themselves be used to mitigate this situation partially, in that microbial gene families that are prevalent and abundant but not yet well-understood can be prioritized for characterization. Likewise microbial communities provide a new source of guilt-by-association information that can be used computationally to generate predictions of gene function [127, 128]. Nevertheless, returning to the field’s microbiological roots may ultimately prove most important in this area: the best biochemical characterizations still derive from culture-based physiology, microbial metabolism, co-culture and interactions, and controlled laboratory environments coupled with high-throughput molecular assays [15, 129, 130].

Studies of the microbiome, both in health and in disease, must continue to integrate population-scale epidemiology with narrow but deep clinical studies in the setting of personalized medicine. In both cases, studies of the body-wide microbiome can be seen as an extension of microbial techniques already used for infectious disease surveillance [131]: rather than waiting to monitor a pathogen’s outbreak in a population or its persistence within an individual, our complete microbial community could be monitored for health maintenance or departures into disease. This is equally true in the integration of microbiome activity with host immune, transcriptional, epigenetic, and clinical state: precision microbial community medicine must rely on host–microbiome interactions as a key component. This will help to identify potential pathogens rapidly [132] and will make it possible to determine the “right” interventions to restore health after dysbiosis, ranging from dietary or lifestyle changes through probiotics into microbially targeted pharmaceuticals [133]. A better understanding of the healthy microbiome must thus approach it as one aspect of deeply monitored personalized health (e.g., [121]) and must integrate population-scale assessment of the microbial community with a well-characterized molecular understanding and analyses of how beneficial community states are maintained body-wide and life-long.

## Abbreviations

FISH: fluorescence in situ hybridization
HMO: human milk oligosaccharides
HMP: Human Microbiome Project
MetaHIT: Metagenomes of the Human Intestinal Tract
SCFA: short chain fatty acid
